# Barriers to sexually transmitted infection testing in New Zealand: a qualitative study

**DOI:** 10.1111/1753-6405.12680

**Published:** 2017-06-29

**Authors:** Hayley J. Denison, Collette Bromhead, Rebecca Grainger, Elaine M. Dennison, Annemarie Jutel

**Affiliations:** 1 School of Biological Sciences, Victoria University of Wellington, New Zealand; 2 College of Health, Massey University, New Zealand; 3 Department of Medicine, University of Otago Wellington, New Zealand; 4 MRC Lifecourse Epidemiology Unit, University of Southampton, United Kingdom; 5 Graduate School of Nursing, Midwifery and Health, Victoria University of Wellington, New Zealand

**Keywords:** sexually transmitted infections, healthcare-seeking behaviour, testing, qualitative, primary care

## Abstract

**Objective:**

To investigate the barriers that prevent or delay people seeking a sexually transmitted infection (STI) test.

**Methods:**

Qualitative in-depth interviews were conducted with 24 university students, who are a group prone to behaviours putting them at risk of STIs, to understand the factors that had prevented or delayed them from going for an STI test in the past. Resulting data were thematically analysed employing a qualitative content analysis method, and a final set of themes identified.

**Results:**

There were three main types of barrier to STI testing. These were: personal (underestimating risk, perceiving STIs as not serious, fear of invasive procedure, self-consciousness in genital examination and being too busy); structural (financial cost of test and clinician attributes and attitude); and social (concern of being stigmatised).

**Conclusions and implications for public health:**

These data will help health providers and policy-makers provide services that minimise barriers and develop effective strategies for improving STI testing rates. The results of this study suggest a holistic approach to encouraging testing is required, which includes addressing personal beliefs, working with healthcare providers to minimise structural barriers and developing initiatives to change social views about STIs.

Sexually transmitted infections (STIs) are a long-standing global health problem with potential serious sequelae including pelvic inflammatory disease, adverse pregnancy outcomes, infertility, rheumatological complications, cancer, organ damage and death.^1^–^4^ Although progress towards control of STIs has been made, for example with antiviral therapy for human immunodeficiency virus (HIV)^5^ and the introduction of the human papillomavirus (HPV) vaccine,^6^ we are still some way from controlling or eradicating these and other pathogens. Of particular concern is increasing rates of antimicrobial resistant gonorrhoea^7^ and the re-emergence of syphilis.^8^

Young people are disproportionately affected by STIs with the majority of infections in the 15–24 year old age group.^9^ University students may be especially vulnerable to STIs. Previous studies indicate that tertiary students engage in multiple high-risk sexual behaviours such as infrequent condom use, sex with multiple partners and casual sex.^10^,^11^ In addition, students typically exhibit high levels of alcohol use,^12^,^13^ which is associated with high-risk sexual behaviour and STI diagnosis.^14^,^15^

Testing and treating infected individuals limits the harm that STIs may cause to the individual and also reduces the potential for transmission to new partners. Regular STI testing is recommended for sexually active young people in many high-income countries.^16^–^18^ However, for this to be effective, the at-risk individuals must know it is recommended and choose to present for testing.

There are many documented barriers to accessing STI prevention and management services. These may be the result of how services are structured, but may also be due to fear, embarrassment, stigma and shame.^19^ To encourage STI testing, health providers and decision makers need to understand in detail the barriers that prevent or delay people from testing.

New Zealand (NZ) provides an interesting case study to investigate barriers to testing for the following reasons. Firstly, NZ has a high incidence rate of chlamydia compared to other high-income countries including the UK, the US and Australia.^20^ In NZ, as in most other high-income countries worldwide, opportunistic testing by clinicians is encouraged but seeking an STI test is usually an individual decision. NZ has a mixed public–private healthcare system, which means STI testing is generally free to those under 22 years old but often attracts a fee for older people who seek testing through their general practitioner (GP). These fees may dissuade individuals at risk of STI from attending for testing. Lastly, there have been no published studies on barriers to STI testing in NZ for a decade. Here, we present data from the qualitative arm of a multi-method study investigating healthcare-seeking behaviour for STI in a university population.

## Methods

In-depth, one-on-one interviews were conducted with students from a university in NZ. Students who had had an STI test at the university clinic and had completed a questionnaire about their visit as part of another phase of this research were recruited by email invitation. Only those who had consented to further contact when they completed the original questionnaire were contacted by the study team. Participants were eligible to take part if they were university students and had presented for an STI test in the past 18 months.

Interviews were held in private rooms on the university campus and were conducted by one researcher (HD), with the exception of the first two interviews where a second researcher (AJ) attended with the participants’ permission. The main interviewer was a female researcher in her early 30s. The participants and interviewer were unknown to each other prior to the interview. Study Information Sheets detailing the background, methodology and confidentiality aspects of the study were provided to the participants prior to the scheduled interview date, and further explanation was offered when the participant attended for their interview. Informed, written consent was obtained before the interview began and participants were given a grocery voucher of small value at the end of the interview as an expression of thanks for their participation.

Privacy and confidentiality were prioritised when designing the study methods. Only the main interviewer knew the participants’ names and email correspondence was deleted following the interviews. Consent forms were kept in a locked file with access restricted to only the main interviewer. The identity of the participants was removed from the data and all electronic information was kept in a password-protected file. Ethical approval was granted by the Victoria University of Wellington Human Ethics Committee (ref: 22110).

The interview was semi-structured and focused on the factors contributing to the decision-making process about going for an STI test. The opening question was: “Why did you go for an STI test?” Open-ended follow-up questions and probes were used to gain further insight into the factors that discouraged/delayed/prevented attending. An iterative process was employed so that questions were informed by previous interviews and the emerging categories.

Each interview was audio recorded and transcribed verbatim. Resulting data were thematically analysed by the authors using NVivo software (version 11), employing a qualitative content analysis method.^21^ Data fragments were assigned codes inductively and a constant comparative approach was taken to ensure codes were used consistently. These codes were grouped to form categories that became the main themes of our analysis. Two researchers (HD and AJ) worked together to identify the final themes and these were tested against the coded transcripts to ensure they were representative of the data. Interviews were continued until theoretical saturation had been reached.

## Results

In total, 24 interviews were conducted, at which data saturation was deemed to have been reached. During recruitment, 57 students who had completed the original questionnaire were invited to participate in an interview. Of these, 30 (53%) responded but five were no longer living in the area and so could not take part in a face-to-face interview, and one was subsequently uncontactable. The participants’ ages ranged from 19 to 32 years, and most were undergraduate students (n=22), with two postgraduate students. There were 16 females, seven males and one genderqueer participant. A range of ethnicities were represented: NZ European (n=16); NZ European/Māori (n=2); NZ European/Other (n=2); British (n=3); and Romanian (n=1). The average length of time for each interview (not including the initial introduction to the study and completing of consent forms) was 40 minutes (24–62 minutes).

From the data, we identified three themes around barriers to STI testing. These included personal barriers (underestimating risk, perceiving STIs as not serious, fear of invasive procedure, self-consciousness in genital examination and being too busy), structural barriers (financial cost of test and clinician attributes and attitude) and barriers related to social condemnation (fear of stigma).

### Personal barriers

#### Underestimating risk

The majority of participants did not think they were at risk of contracting an STI. Even those who routinely went for STI tests said that they did not expect to have an STI, but just wanted confirmation of a clean bill of health: *“I try to go for them quite regularly, I don’t ever think I have one I just like to know that I definitely don’t. I generally will go if I’ve had a new partner, even if I don’t think that they’ve got anything.”* – Interview 7 (female, 21 years)

Personal risk assessments were common. Many participants described the process of weighing up evidence to estimate the possibility of having acquired an STI when making the decision to go for an STI test. Indicators included the number of recent sexual partners, familiarity with those sexual partners, and past sexual behaviour of the sexual partners. As one participant explained when asked whether he would seek an STI test after unprotected sex with a new partner: *“It depends who I had a one night stand with. If I knew the person then I probably would put it off more but if I didn’t know the person then I’d go, yep.”* – Interview 16 (male, 21 years)

Several participants, both male and female, indicated that they felt they would be able to tell if a sexual partner was likely to have an STI: *“It’s awful but you can kind of see it from how people act and how they’re dressed if they have slept with a lot of people or if they’re more reserved, etc.”* – Interview 13 (male, 21 years)

There was suggestion that this risk assessing was at least in some part influenced by experience of the health professional conducting a risk assessment when deciding whether to include a HIV test in the screen. If the health professional thought they were low risk for HIV, some participants used this as justification for not getting a HIV test at any time, and more generally as confirmation that they were not a high-risk person in terms of their sexual behaviour:

*“I was asking ‘What is this testing for?’ and she sort of said it’s the more low-level ones and they didn’t have to do a blood test unless you thought you might have contracted one of the serious ones like AIDS. So I haven’t had a blood test or really thought there was a need to. Again, that would be one of those ones that if you thought there was a need to, that’s when I’d probably do it, but otherwise it seems to be a bit of a hassle.” –* Interview 13 (male, 21 years)

#### Perceiving STIs as not serious

Although many participants mentioned infertility as a significant concern that encouraged STI testing, there was a general feeling that chlamydia and gonorrhoea were not serious infections. Syphilis and HIV were viewed as serious, but so rare that they were not to be a source of worry: *“I wasn’t concerned if I had [an STI], I didn’t think I would have one but if I had one I wasn’t concerned because I didn’t think it would have been a bad one. It would have been something like chlamydia.”* – Interview 13 (male, 21 years)

The lack of concern about contracting chlamydia or gonorrhoea was at least partly because they are treatable infections: *“Well like I don’t panic much about stuff like that because unless it’s something very serious you can do something about it once you catch it.”* – Interview 3 (female, 20 years)

A common narrative among the participants was that as they got older, they had come to realise the seriousness of STIs and thus become more responsible in their sexual and testing behaviour: *“If I do have unprotected sex with anyone new I always go and get a check afterwards, I’ve just done that, but when I was a lot younger I didn’t. I was very badly behaved in that respect so I guess the older I get the more aware I am of what’s out there and I do try and protect myself as much as possible, but sometimes things happen you know.”* – Interview 2 (female, 23 years)

#### Fear of invasive procedure

Participants reported having very little understanding of what testing would involve prior to their first STI test. Male participants in particular had higher levels of fear and anxiety surrounding both the discussion with the health professional and the test itself. Both male and female participants had assumed that the process would involve a physical examination and invasive procedure. This made them nervous, and several males reported that this was a contributory factor for not seeking an STI test in the past.

Interviewer: *“Was there anything that put you off going for a test?”*

Participant: *“It was mainly stories I’ve heard from people, like about the cotton bud. It made it sound real painful and that it would just be a really terrible time.”* Interview 16 (male, 21 years)

This uncertainty was not aided by different care providers adopting different protocols for asymptomatic STI testing. Several males reported experiencing a range of testing procedures across different care providers for repeat STI checks, including clinician-taken swabs, first-void urine tests and blood tests. As one male participant explained:

*“Every time there’s been a different process to go through to get the check. I’ve had the urine sample, the swab and blood tests as well and it hasn’t been any kind of pattern to what, yeah, and so even now I’m still like ‘What is going to happen if I do go and get a check?’” –* Interview 15 (male, 23 years)

Even after having one or several STI tests, participants said they had little understanding of the testing process and of what infections were being tested for:

*“I didn’t really know what it was that was covered by the tests, I don’t remember having any knowledge of that and I remember walking out of there and I didn’t question, I didn’t really ask heaps of questions. I remember walking out of there going ‘Oh I’m glad that they didn’t have to stick that thing down my urethra’.” –* Interview 18 (male, 24 years)

This was especially true with regards to HIV. Around half the participants said they did not know a blood draw was required to do a HIV test or had not known before they’d had a HIV test. Although the participants viewed HIV as a very serious infection, more than half had never had a HIV test or were unsure if they had been tested for HIV, despite having had a chlamydia and gonorrhoea test. For example, when asked whether she had ever had a HIV test, one female responded:

*“A couple of times they have sent me down as part of the STI testing to have blood drawn and get urine samples taken and those have been analysed and they’ve come back clear, so they’ve possibly tested at the same time for HIV but I wouldn’t know.” –* Interview 4 (female, 23 years)

When questioned about whether a blood draw would deter them from testing, only a few participants said that it would.

#### Self-consciousness in genital examination

Many participants reported being extremely nervous before their first STI test. Having to undress and be examined worried many participants, and the general assumption prior to a first STI test was that a physical examination was a necessary part of the process: *“It’s embarrassing like having to take your pants off and having the doctor like, you know, fondling your stuff, it’s like not the best to be honest.”* – Interview 20 (male, 23 years)

Males unanimously preferred the self-taken urine test to being examined by a doctor, but the attitude towards self-swabs among the female participants was mixed. Some were grateful that they would not need to show their genitalia to a health professional; whereas others were concerned that they would not perform the self-swab correctly, producing an erroneous result, or that it would hurt. As one woman who had recently had an STI test explained: *“It was a self-swab, like you did it yourself… I like that better because I didn’t have to panic about anything else. I didn’t have to be like ‘Do I look weird down there?’ So it was easy, it took like five minutes, not even that, so that was good.”* – Interview 3 (female, 20)

Another participant explained why she opted for her STI test to be done by the clinician: *“I was worried that I’d do it wrong. It’s like that when you first use tampons or something you’re like ‘What am I doing?’ So that was probably my only concern, or like yeah, just worried that maybe you won’t do it right and the tests won’t be accurate or whatever.”* – Interview 7 (female, 21 years)

#### Too busy

Another factor that was mentioned in relation to not seeking an STI test or putting off testing was simply being too busy to attend, indicating that it was not a priority: *“It’s one of those things that you have to just try and keep up with, but you know life gets in the way sometimes.”* – Interview 22 (female, 30 years). Reasons for being too busy included university assignments and tests, paid work and social commitments.

A few participants mentioned the speed of the appointment when explaining why their STI test experience had been good, suggesting this is an important factor for some young people: *“It’s not like it takes a long time, it’s just ten minutes in and out, like yep, you just wait for a call and like I reckon if you get tested more often than not you’re going to not get a call which is always nice*.*”* – Interview 11 (female, 19 years)

Two participants said it took them a few days to go in for treatment after being contacted by their doctor about their test results because they were too busy to attend straight away. However, both reported avoiding sex until after they had completed their treatment.

### Structural barriers

#### Financial cost of STI test

The cost of STI testing was considered a potential barrier by the participants in our study. The university clinic provides free STI tests to domestic students; therefore the majority of participants in this study had not needed to pay for their tests, which they viewed as conducive to their attending: *“Like with my current income and financial position I probably couldn’t afford to go as often as I did if it wasn’t free.”* – Interview 10 (female, 21 years)

Many stated that they simply would not have an STI test in the absence of symptoms if they were required to pay, and several pondered aloud what they would do after they graduated and no longer had access to the university clinic. However, it was interesting to note that several participants had previously paid for an STI test elsewhere (for example, when away from the university during holidays) in the absence of symptoms, usually after engaging in a sexual situation they perceived to be high risk.

#### Clinician attributes and attitude

Although many participants expressed a preference for a same-sex health professional, most said they would not be deterred from having an STI test if a health professional of their preferred gender was not available:

*“I don’t care if it’s a male doctor; well I don’t care too much if it’s a male doctor that performs those kind of procedures. In general, I would prefer it if it was a woman but, for example, if I couldn’t have an appointment with a woman doctor until the next week and there was a male doctor who was available that day then I’d go with the male doctor, and if he’s not comfortable doing swabs he can get a nurse to do it, but I mean he’s a doctor he should be, yep. So yeah, the gender of the doctor sometimes has an impact.”* – Interview 6 (female, 32 years)

Only one (heterosexual male) participant spoke about not wanting to be examined by a female doctor because it would be difficult to disassociate that from a sexual situation:

*“I didn’t really want her to have a look because you know, it’s just an uncomfortable situation I guess. Just because, I don’t know, you have that like male female sort of thing like it feels kind of wrong, the situation, it’s hard to displace the fact that they’re a female touching your penis from a sexual situation I guess. Whereas if it’s a male it’s like ‘ok, this is a doctor, this is fine’.” –* Interview 13 (male, 21 years)

Men, before their appointments, tended to expect that the nurses would be judgemental or morally disapproving of their behaviour and that the consultation would be awkward. Once having attended, they found they had generally felt comfortable discussing sexual health with the healthcare professional. A few participants did report a negative attitude during an STI test consultation but this hadn’t seemed to have put them off seeking an STI test subsequently. Rather, they said they would simply visit a different clinician next time.

Other health-service related barriers included: difficulty booking a timely appointment and the inconvenience of having to visit a separate location for a blood draw in some cases. Although around one-third of participants described dissatisfaction with having to wait for an available appointment, it did not seem to deter individuals from attending. However, it does have implications for onwards transmission in the meantime, as one participant described: *“I feel the waiting times are a little bit ridiculous, it’s usually like at least a week which is a little bit stupid because you can do anything within the span of a week.”* – Interview 9 (female, 27 years)

Concerns about privacy and confidentiality in relation to the health provider were not frequently mentioned. A few participants mentioned not using their family doctor for sexual health issues, but preferring to go to the university clinic. These individuals did not think the doctor would breach their confidentiality, but rather that there was more anonymity with the student clinic because they were less likely to be seen in the waiting room by someone known to their family or whānau: *“Yeah with the family GP it is definitely like ‘Oh what if someone sees me, what if someone walks in, a family friend, a school friend’, yep. Whereas at uni, it’s sort of like, it’s a bit different, it’s more casual almost.”* – Interview 24 (male, 20 years)

### Social barriers

#### Concern of being stigmatised

Stigma, which can be defined as an attribute, behaviour, or reputation that has a negative effect on how a person is perceived,^22^ emerged as an important factor influencing STI testing behaviour:

*“STIs are very much stigmatised, I think that’s what the big issue is and so I think that’s why I don’t think people go and get tested as much because they’re like ‘Oh my god, I’ve got chlamydia and that’s so embarrassing’.”* – Interview 3 (female, 20 years)

It was clear in the way many of the participants spoke that they were aware of the stigma associated with STI testing and so would avoid testing or not disclose their testing activities widely: *“They might think that you either have an STI or that you might be considered like a little bit of a slut because you’ve been sleeping with other people and not using protection and stuff like that. So I wouldn’t tell anybody but I guess with your close friends you know they probably know that it’s just like routine, yeah.”* – Interview 21 (female, 21 years)

Though some participants actively challenged this stigma: *“It’s no different to any other sort of check-up when you go to the doctors. I think if anyone come up to me about why are you getting a check-up, it’s like well I’m the one’s that’s looking after myself and making sure that my partner’s healthy as well.”* – Interview 24 (male, 20 years)

It should be noted that the stigma discussed by the participants was perceived stigma, rather than actual stigma, as very few had stories of actually being discriminated against due to STI testing or being diagnosed with an STI. The opinions that most participants feared were those of ex-, current or future partners, not those of friends or acquaintances, and one of the most fear-inducing consequences of having contracted an STI was the need to inform current and recent sexual partners.

It was encouraging that a small number of participants felt that stigma about STIs was reducing: *“I think like a lot of the stigmas around STIs are disappearing and that’s really cool. So I think like everyone’s kind of more aware that it’s a lot easier to contract, it’s not that you have to sleep with a hundred different people to get one.”* – Interview 2 (female, 23 years)

## Discussion

This study found several barriers to STI testing, which can be broadly grouped into three overall themes: personal barriers, structural barriers and social barriers.

Most participants did not feel they were at risk of contracting STIs and, even after deciding to have an STI test, very few believed they would receive a positive STI test result. This lack of risk perception has been reported in other studies.^23^–^27^ Participants generally felt that they could judge for themselves whether a sexual partner or potential partner was ‘clean’ or not. Participants would look for indicators of ‘dirtiness’, including the way individuals dressed and acted. This attitude reflected that found among male students in a UK university who maintained that they could avoid chlamydia by being wary of ‘risky types’ of women.^28^ A sense of invulnerability is potentially dangerous as people will not take the necessary steps to protect themselves if they do not feel at risk. Making people aware of their own risk and the severity of STIs may be one way to encourage STI testing in this population.

Many participants in this study had never had an HIV test, even if they had been tested for chlamydia and gonorrhoea. Similar findings have been reported from other studies of this age group and it is suggested this is because young people have not been as exposed to the high volume of public health messages about HIV as previous generations were during the initial stages of the epidemic in the 1980s and ’90s, and they have seen less HIV-associated mortality due to improvements in antiretroviral therapy.^29^

As reported by other studies, we found that prior to a first test, males expected the STI test to involve a urethral swab and be a painful experience.^28^,^30^–^32^ Although this expectation had initially prevented some males from attending for an STI test, most participants reported that these fears were alleviated at their first consultation and they became more proactive about re-attending for STI testing. Current laboratory diagnostic techniques allow for urine tests to be used for *C. trachomatis* and *N. gonorrhoeae* testing in asymptomatic men. However, several males in this study reported experiencing different types of tests on different occasions (even when asymptomatic), which led to confusion and renewed anxiety about what to expect. This suggests that standardising care so that only urine tests are used for asymptomatic males may result in less confusion and increased healthcare-seeking behaviours.

Being too busy was a reason for some participants to put off seeking an STI test. This finding indicates that STI testing isn’t a priority for these individuals and links with the sub-themes of underestimating risk or perceiving STIs as not serious. Offering testing as part of a clinical consultation for another reason may be one method of circumnavigating this issue; if the individual has already attended for a health issue they do see as a priority. Opportunistic testing for chlamydia is recommended in NZ for all sexually active people aged under 25 years.^18^ The findings of this study support this recommendation.

The cost of STI testing was frequently mentioned as a barrier. However, there seemed to be a dichotomy between regular low-risk testing and testing because they perceived to be a problem that was seen as worth paying a fee. Given that most individuals fail to accurately assess their risk, this has serious implications for identifying untreated infection in the community. In NZ, sexual health clinics are publically funded and provide free tests and treatment for STIs. However, GP visits are only partly subsidised by the government, meaning patients are often required to make a co-payment for services. Funding for free sexual health consultations with GPs is irregular and depends on the District Health Board, individual Primary Health Organisation and practice decisions. The university clinic from where the participants of this study were recruited provided free sexual health consultations for domestic, but not international, students. Previous research in NZ showed that introducing free sexual health GP consultations for under-25-year-olds significantly increased the number of people attending for an STI test, as well as the number of chlamydia infections diagnosed.^33^ Health providers need to be aware that the fees associated with STI testing may reduce regular check-ups in the absence of symptoms, and aim to provide free services as widely as possible.

Additional structural barriers to STI testing included difficulty booking a timely appointment, having a separate location for blood draws, and the gender of the health professional. Although these factors were offered as barriers, in many cases the participants had still chosen to present for STI testing. Patient–doctor confidentiality was not a big concern for the individuals in this study, which is contrary to findings from several previous studies of young people in the US and Australia.^30^,^34^,^35^ This finding may represent a cultural difference, or may be due to our sample being drawn from a clinical site, where those who do not trust the confidentiality of the patient–doctor relationship would be less likely to be present.

Similar to other studies,^34^,^36^,^37^ fear of stigmatisation seemed to be an important factor preventing seeking STI testing for these participants. Stigma may negatively affect healthcare-seeking behaviour for STI testing because seeking an STI test suggests that socially undesirable behaviour has occurred,^38^ and this presents a threat to people’s social identities.^39^ Male participants in this study appeared to be more concerned about being stigmatised if they were seen to be requesting an STI test than female participants.

Barriers to STI testing are understudied in NZ. Rose et al. carried out focus groups with 16 to 24-year-olds and health professionals to find ways to encourage chlamydia testing.^40^ The study identified several reasons as to why young New Zealanders would not seek chlamydia testing, including fear, stigma, denial of personal risk and lack of knowledge, which reflect some of the barriers found in the current study. This suggests that there has been little change in the perceived barriers to STI testing over the past decade in NZ, indicating that more needs to be done to alleviate these obstacles to testing.

There were some potential limitations of the current study. Firstly, the interview schedule was not piloted before the interviews began. Piloting study questions may have provided us with insight into which questions best facilitated disclosure of relevant information by participants. However, because we planned *a priori* for our interview schedule to be flexible and evolving (whereby questions were omitted and added as categories emerged and themes became clearer), we felt this would be an inefficient use of time and resources. University students are not representative of all young people and barriers to testing may be different in non-student populations. That said, comparisons with similar studies (as outlined above) indicate that there are similarities with other groups, so transferability of findings may be valid. In addition, those that agreed to be interviewed may have more of an interest in this topic and so may have different opinions and experiences to those who declined to participate. While there was some ethnic diversity within the study sample, only two participants identified as Māori and no participant identified as Pacific. The reasons for this are unclear, though one possible explanation could be that cultural differences in the acceptability of discussing sexual issues with a stranger may have deterred participation. Māori and Pacific peoples experience a higher burden of infection than NZ Europeans.^20^ Unfortunately, the low number of Māori and Pacific peoples in this study limits the ability to draw any valid conclusions about specific issues with regards to healthcare-seeking behaviour for STI testing for these people. Understanding the specific needs and barriers of Māori and Pacific peoples is necessary to enact evidence-based changes leading to more equitable sexual health within NZ.

It is important to note that these individuals had access to an on-campus health service that offered free STI tests to domestic students, which may have reduced some of the usual barriers to testing. In addition, the participants in this study were recruited through a clinical site and all had experienced an STI test at least once, therefore the views of those who have never had an STI test are not represented in these data. These may be the people for whom barriers exert a particularly strong effect, or there may be different barriers for the people not represented in this study. That said, Richardson et al. interviewed 14 young people aged 16–24 years who had turned down chlamydia testing as part of the chlamydia screening program in England and identified similar themes: stigma, embarrassment, perception of risk and beliefs of what the test involves.^37^

This paper provides information on what prevents or discourages STI testing in a NZ population. Such information is helpful in the generation of interventions to promote STI testing uptake. Traditionally, efforts have targeted individual level factors associated with STI risk, but this study supports the idea that, to be effective, they will also need to address higher-level factors such as social and cultural conditions that also influence healthcare-seeking behaviours.^41^ With this in mind, a holistic approach needs to be taken, targeting individual-level factors such as personal beliefs, working with healthcare providers to minimise structural barriers, and developing polices and initiatives designed to change social beliefs about STIs. specific interventions could include providing more information about the testing procedure, perhaps emulating ways this has been done previously in NZ with condom promotion.^42^ In addition, making sexual health consultations free for everyone and developing initiatives that encourage open discussion of STIs to reduce the stigma surrounding them would be key areas of focus.
